# Enzymatic Synthesis of Structured Lipids Enriched with Medium- and Long-Chain Triacylglycerols via Pickering Emulsion-Assisted Interfacial Catalysis: A Preliminary Exploration

**DOI:** 10.3390/molecules29040915

**Published:** 2024-02-19

**Authors:** Zhe Dong, Ziheng Cui, Jun Jin, Xinyi Cheng, Gangcheng Wu, Xingguo Wang, Qingzhe Jin

**Affiliations:** 1 State Key Lab of Food Science and Resources, School of Food Science and Technology, Jiangnan University, Wuxi 214122, China; dongzhehehe@163.com (Z.D.); junjin@jiangnan.edu.cn (J.J.); chengxinyi2018@163.com (X.C.); gangcheng.wu@jiangnan.edu.cn (G.W.); xingguow@jiangnan.edu.cn (X.W.); 2 National Energy R&D Center for Biorefinery, Beijing University of Chemical Technology, Beijing 100029, China; cuileft@gmail.com

**Keywords:** medium- and long-chain triacylglycerol, pickering emulsion interfacial catalysis, enzymatic trans-esterification, tunnel analysis, molecular dynamics simulation

## Abstract

Medium- and long-chain triacylglycerol (MLCT), as a novel functional lipid, is valuable due to its special nutritional properties. Its low content in natural resources and inefficient synthesis during preparation have limited its practical applications. In this study, we developed an effective Pickering emulsion interfacial catalysis system (PE system) for the enzymatic synthesis of MLCT by trans-esterification. Lipase NS 40086 served simultaneously as a catalyst and a solid emulsifier to stabilize the Pickering emulsion. Benefitting from the sufficient oil–water interface, the obtained PE system exhibited outstanding catalytic efficiency, achieving 77.5% of MLCT content within 30 min, 26% higher than that of a water-free system. The *K*_m_ value (0.259 mM) and activation energy (14.45 kJ mol^−^^1^) were 6.8-fold and 1.6-fold lower than those of the water-free system, respectively. The kinetic parameters as well as the molecular dynamics simulation and the tunnel analysis implied that the oil–water interface enhanced the binding between substrate and lipase and thus boosted catalytic efficiency. The conformational changes in the lipase were further explored by FT-IR. This method could give a novel strategy for enhancing lipase activity and the design of efficient catalytic systems to produce added-value lipids. This work will open a new methodology for the enzymatic synthesis of structured lipids.

## 1. Introduction

Medium- and long-chain triacylglycerol (MLCT) is a novel functional lipid containing long-chain fatty acids (LCFAs) and medium-chain fatty acids (MCFAs) in one glycerol backbone. Many studies have demonstrated the beneficial physiological effects of MLCT in animal experiments and clinical trials, such as body fat control and gut microbiota and lipid metabolism regulation [1,2]. However, these desirable structured lipids are present in insufficient amounts in natural lipids, limiting their large-scale production and commercial applications. The development of straightforward preparation methods for structured lipids is a necessity and essential for pervading all aspects of food application.

Enzymatic synthesis is gaining growing popularity due to mild reaction conditions and remarkable specificity. Lipase is one of the most advanced and typically utilized enzymes in the enzymatic synthesis of MLCT [3,4,5]. In general, lipase is dispersed in the aqueous phase, but the involved viscous substrates are mostly oil-soluble, indicating that this reaction is a biphasic catalytic reaction process. These biphasic reactions always suffer from low efficiency due to the low mass transfer and high interfacial energy of immiscible phases [6]. To circumvent this issue, organic solvents are added into the system to reduce the viscosity, while the subsequent removal process remains laborious. Although ultrasonic treatment or microwave irradiation could facilitate the interaction between the lipase and the substrate, it faces limitations related to the confined interfacial area and the potential damage or even denaturation of lipase from ultrasound [7,8,9]. Therefore, further development of new and effective strategies for preparing MLCT is highly desirable.

Recently, Pickering emulsions (with solid particles as emulsifiers) hold great promise in heterogeneous catalysis, especially for biphasic reactions due to their high interfacial area, stable compartmentalized structure, and easy separation and recycling of particles [10]. Pickering emulsion-assisted interfacial catalysis, in which solid particles serve simultaneously as emulsifiers and catalysts at the interface of two immiscible phases, has become an attractive platform for the green transformation of chemicals and foods [11,12,13]. An example of Pickering emulsion-assisted interfacial (PE system) transphosphatidylation has been reported for the enzymatic synthesis of phosphatidylserine [14]. The catalytic efficiency in the PE system was 21-fold higher than that of the free enzyme due to a sufficient oil–water interface at the active sites of the enzyme. Moreover, this novel reaction system has been applied in continuous flow processes [15] and cascade reactions for the hydrolysis of high-acid-value oils [16,17]. To this end, it is promising to design a productive reaction strategy based on PE interfacial catalysis for the enzymatic synthesis of MLCT. Meanwhile, extensive research has focused on the preparation of a stable Pickering emulsion with multifarious proteins and the characterization of the emulsion. Enzymes, distinctive proteins, are characterized primarily by their catalytic property and specific structures. However, there is a lack of understanding of the relationship between enzyme structure and catalytic activity in the PE reaction system, in particular its molecular-scale dynamics.

**Scheme 1:**
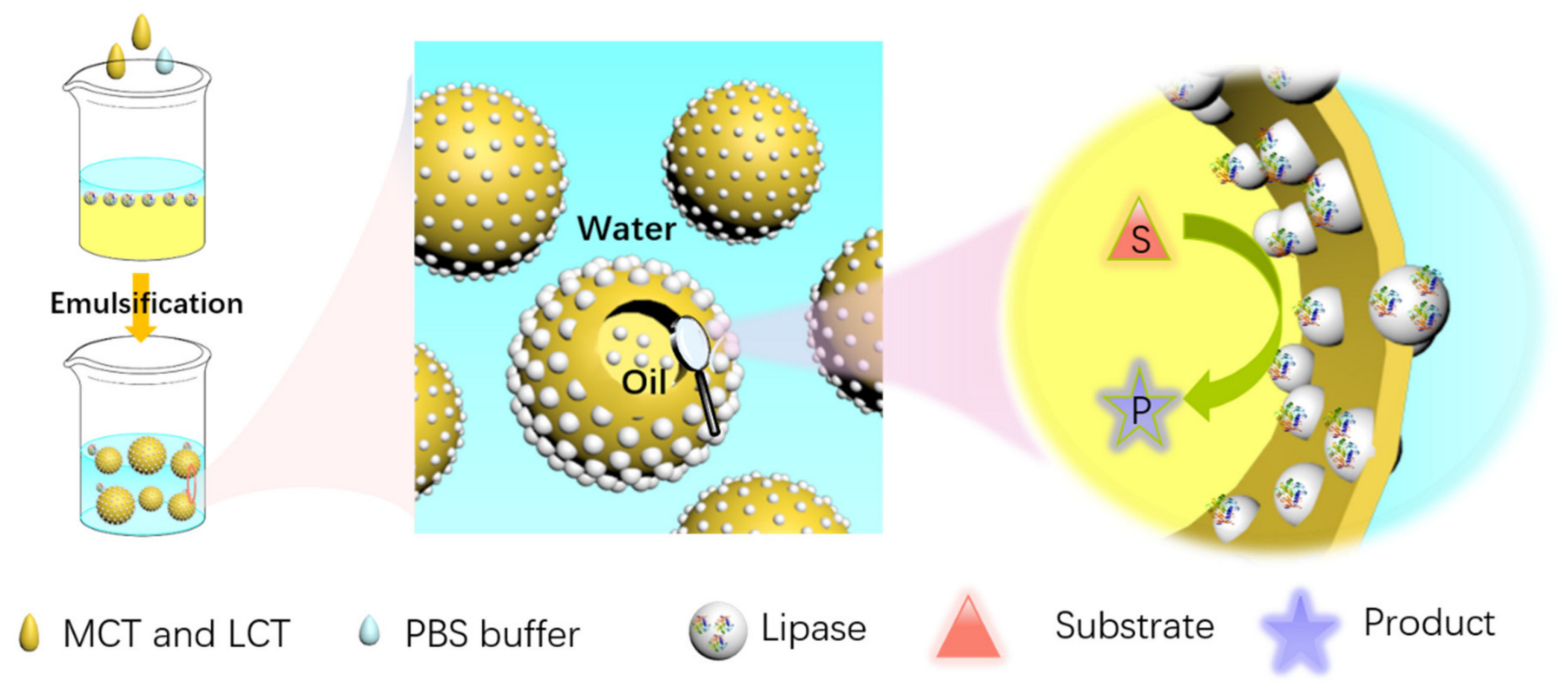
Construction of Pickering emulsion interfacial catalysis.

Herein, we demonstrate a novel strategy for the enzymatic synthesis of MLCT through the construction of a Pickering emulsion system (PE system). In this proposed method, the substrates medium-chain triacylglycerol (MCT) and camellia oil (long-chain triacylglycerol, LCT) were considered as the oil phase, and an adequate amount of phosphate-buffered solution buffer was added as the aqueous phase. During the synthesis process, the immobilized lipase NS 40086 particles served simultaneously as emulsifiers to stabilize the Pickering emulsion and as catalysts to bring about the trans-esterification between MCT and LCT (Scheme 1). The key parameters of the PE system were optimized, including the ratio of oil to water, the particle loading amount, and the temperature. Significantly, the conformational changes in the lipase were characterized by FT-IR, a molecular dynamics (MD) simulation, and a tunnel analysis at the molecular level. Moreover, this study compared the kinetics and thermodynamics of a traditional system (water-free system) and the PE system as well as their triacylglycerol (TAG) compositions using ultra-performance liquid chromatography and a quadrupole time-of-flight mass spectrometer (UPLC-Q-TOF-MS).

## 2. Results and Discussion

### 2.1. Characterization of Pickering Emulsion

**Figure 1:**
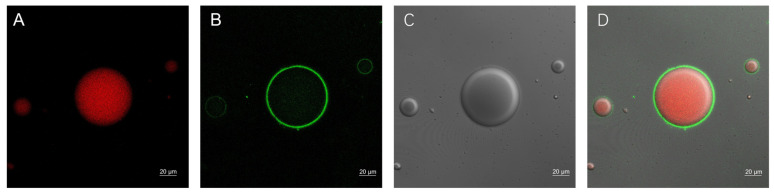
The CLSM images of (**A**) the oil phase stained by Nile red; (**B**) NS 40086 stained by FITC-I; and (**C**) bright field (**D**) merge. (Measuring scale is 20 μm).

Figure 1A–D display the CLSM images of the Pickering emulsion system of NS 40086. The biocatalyst lipase NS 40086 is labeled with FITC-I, and the oil phase is stained with Nile red. The green circular ring in Figure 1B representing NS 40086 is indeed located at the interface. The red droplet shows that this emulsion is an oil-in-water emulsion. This result indicates that the Pickering emulsion has been established with substrates and biocatalyst.

### 2.2. Fatty Acids and TAG Profiles of MCT and Camellia Oil

The structured MLCT was synthesized by the trans-esterification of MCT and camellia oil. Considering that hydrolysis can occur in ways besides trans-esterification, there were free fatty acids, diacylglycerols, and monoglycerides. Thus, TLC was used to separate those lipids, and the pure triglycerides were acquired for the following detection. The produced triglyceride, MLCT, was then detected by HPLC. The fatty acid profile of the substrates was determined by GC and is shown in Table S1. The fatty acid composition of the MCT was caprylic acid (55.11%) and octanoic acid (44.89%), which were all MCFAs. The major fatty acids of the camellia oil were LCFAs, which include palmitic acid (8.77%), oleic acid (80.16%), and linoleic acid (6.34%). The identified TAGs of these substrates are displayed in Table S2. The main TAGs in the MCT were 8:0/8:0/8:0 (ECN 24, 24.95%), 8:0/10:0/8:0 (ECN 26, 40.00%), 8:0/10:0/10:0 (ECN 28, 29.05%), and 10:0/10:0/10:0 (ECN 30, 5.95%), which were MMM-type TAGs. In the camellia oil, the predominant TAGs were 18:1/18:1/18:1 (ECN 48, 58.70%), 18:1/16:0/18:1 (ECN 48, 19.58%), and 18:1/18:2/18:1 (ECN 46, 10.84%). The oil had a low amount of 18:1/16:0/18:2 (ECN 46, 4.38%) and 18:1/18:0/18:1 (ECN 50, 4.08%). Therefore, this camellia oil was enriched in LLL-type TAGs and a good raw source for the synthesis of MLCT. The inter-esterified mixture was composed of MMM-, MML-, MLL-, and LLL-type TAGs (including their isomers MLM and LML). In this work, the reaction conversion was referred to as the MLCT content, which is the sum of both MML- and MLL-type TAGs.

### 2.3. Optimization of Synthesis Conditions for Structured MLCT

#### 2.3.1. Effect of Weight Ratio of MCT to Camellia Oil on MLCT Content

Given the fact that enzymatic trans-esterification is reversible, the conversion of substrates through this process would be incomplete [18], and the composition of said substrates could affect their incomplete conversion, indicating that the proper ratio of substrates is of great significance. Thus, the effect of the weight ratio of the MCT to the camellia oil was optimized. As shown in Figure S1A, when the MCT/camellia oil weight ratio increased from 0.5:1 to 1:1, the content of MLCT increased slightly from 73.84% to 75.76%. The content of MLL-type TAGs also increased from 28.8% to 31.2%. However, a further increase in the weight ratio resulted in a significant decline in MLCT content. Taking the higher content of MLCT and MLL-type structured lipids into account, together with the cost of the oil, the substrate weight ratio of 1:1 was selected for further investigation

#### 2.3.2. Effect of Enzyme Dosage on MLCT Content

**Figure 2:**
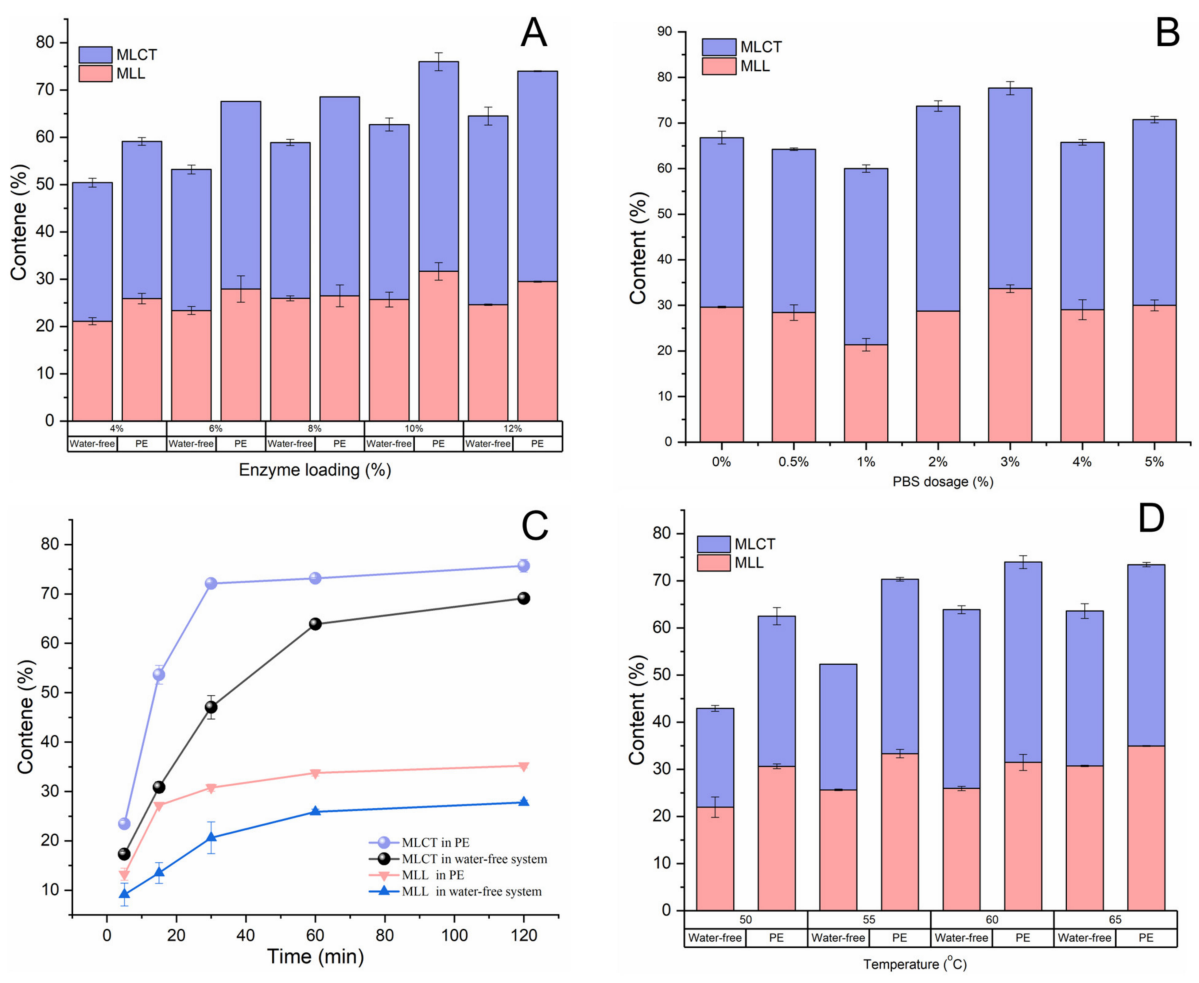
(**A**) Effect of enzyme loading on the MLCT content; (**B**) effect of PBS dosage on the MLCT content. Reaction conditions: substrates ratio, 1:1; enzyme loading, 10%; temperature, 60 °C; and reaction time, 2 h. (**C**)Time process of MLCT synthesis; and (**D**) effect of temperature on the MLCT content. Reaction conditions: substrates ratio, 1:1; enzyme loading, 10%; and reaction time, 2 h.

In an enzymatic reaction, a higher amount of enzyme loading could drastically lower the reaction activation energy and enhance the reaction’s efficiency. Meanwhile, in our study, the enzyme (NS 40086) served simultaneously as an emulsifier to stabilize the Pickering emulsion in the PE system. Thus, the effect of enzyme dosage from 4% to 12% on the MLCT content was investigated. With the successive increase in enzyme dosage from 4% to 10%, the content of MLCT showed a gradual increase from 58.5% to 76.6% in the PE system (Figure 2A). A similar trend was observed in the content of MLL-type TAGs. The PE system achieved a higher MLCT content than the water-free system at each enzyme loading quantity. Considering the cost of the enzyme, the selected enzyme dosage was set to 10%.

#### 2.3.3. Effect of PBS Dosage and pH on MLCT Content

The ratio of water to oil is a fundamental factor for the construction of a stable and efficient PE system. In this kind of reaction, the appropriate amount of PBS dosage can improve the oil–water interface for the lipase and, thus, promote the enzyme’s catalytic performance, while a higher content of PBS leads to a side reaction, such as the hydrolysis of TAGs. The effect of PBS dosage on the yield of MLCT was investigated in our study. As shown in Figure 2B, the content of MLCT showed little change, and even a slight decline was observed when the PBS dosage was less than 2%. This might have resulted from the ineffective dispersal of the water phase with a smaller PBS dosage, which failed to form the oil–water interface. Meanwhile, the MLCT content increased significantly from 66.7% to 77.5% when the PBS dosage increased from 1% to 3% (*w*/*w*, buffer to total weight). Compared with the water-free reaction system (0% PBS dosage), the MLCT content in the PE system was significantly higher. Thus, we envisioned that the oil–water interface was formed by the PBS and the substrate TAGs, and the biocatalyst lipase NS 40086 was located at the interface, which promoted the lid opening of the lipase. The addition of a PBS dosage higher than 3% did not contribute to a further accumulation of MLCT, and a distinct decrease in the MLCT content was observed. Therefore, the optimized PBS dosage was 3%.

There are some similarities between Pickering emulsion interfacial catalysis and water’s effect in an organic reaction. As it has been reported in some reviews, the key of all reactions dealing with water is that water is viewed as a reaction medium, and the amount of water has little effect on a given reaction’s efficiency or selectivity [19,20]. In this study, the content of MLCT varied with the different PBS dosages (“water”), as shown in Figure 1B. Thus, this study did not fall in the above-mentioned cases and belonged to the Pickering emulsion interfacial catalysis category instead.

The time process of these two different systems was completed under the optimized conditions. As depicted in Figure 2C, the PE system presented a faster reaction rate, with an MLCT content of up to 70% at 30 min, which was 26% higher than that of the water-free system. By prolonging the reaction time to 120 min, little significant increase in the conversion to MLCT in the PE system was observed, indicating that the reaction achieved equilibrium within about 30 min. However, only a 45% conversion was achieved in the water-free system within 30 min, exhibiting a gradual increase in the whole 120 min timeframe. Despite a demanding rise, the MLCT content was invariably less than that of the PE system even at 4 h, revealing that the additional water–oil interface in the PE system may have activated and maximized lipase activity compared to the water-free system.

The effect of the pH value of PBS on the MLCT content was also evaluated (Figure S1B). The MLCT content was maintained at 72% with different PBS solutions of various pH values. It could be seen that the pH value had little effect on the performance of lipase NS 40086 in the PE reaction system. The highest content of MLCT was achieved at a pH of 7.0. Thus, the pH value of the PBS was set to 7.0 in the following study.

#### 2.3.4. Effect of Reaction Temperature on MLCT Content

In general, reaction efficiency is positively associated with reaction temperature. A higher reaction temperature could promote the reaction rate but harms lipase activity. Therefore, the effect of the reaction temperature on the MLCT content was investigated in both the water-free system and the PE system in our study. As shown in Figure 2D, the MLCT content in the PE system was always higher than that in the water-free system at each temperature. When the reaction temperature was 50 °C, the MLCT content in the PE system reached 63.7%, 22.6% higher than that in the water-free system (41.1%). The highest MLCT content (74.9%) was gained at a temperature of 60 °C in the PE system. Interestingly, the highest MLCT content in the water-free system was 62% at a temperature of 60 °C. At the temperature of 50 °C, the PE system achieved an MLCT content of 63.7%. This result indicated that lipase NS 40086 in the PE system could enhance the reaction rate and lower the reaction temperature, resulting in a higher MLCT content at each temperature tested. We deduced that the improvement in lipase activity in the PE system contributed to the oil–water interface in boosting the lid opening of the lipase, bringing full access to the catalytic active site. The relationship between the reaction rate and the temperature is further explored in one of the following sections, Reaction Kinetics.

#### 2.3.5. Structural Characterization of the Enzyme

**Figure 3:**
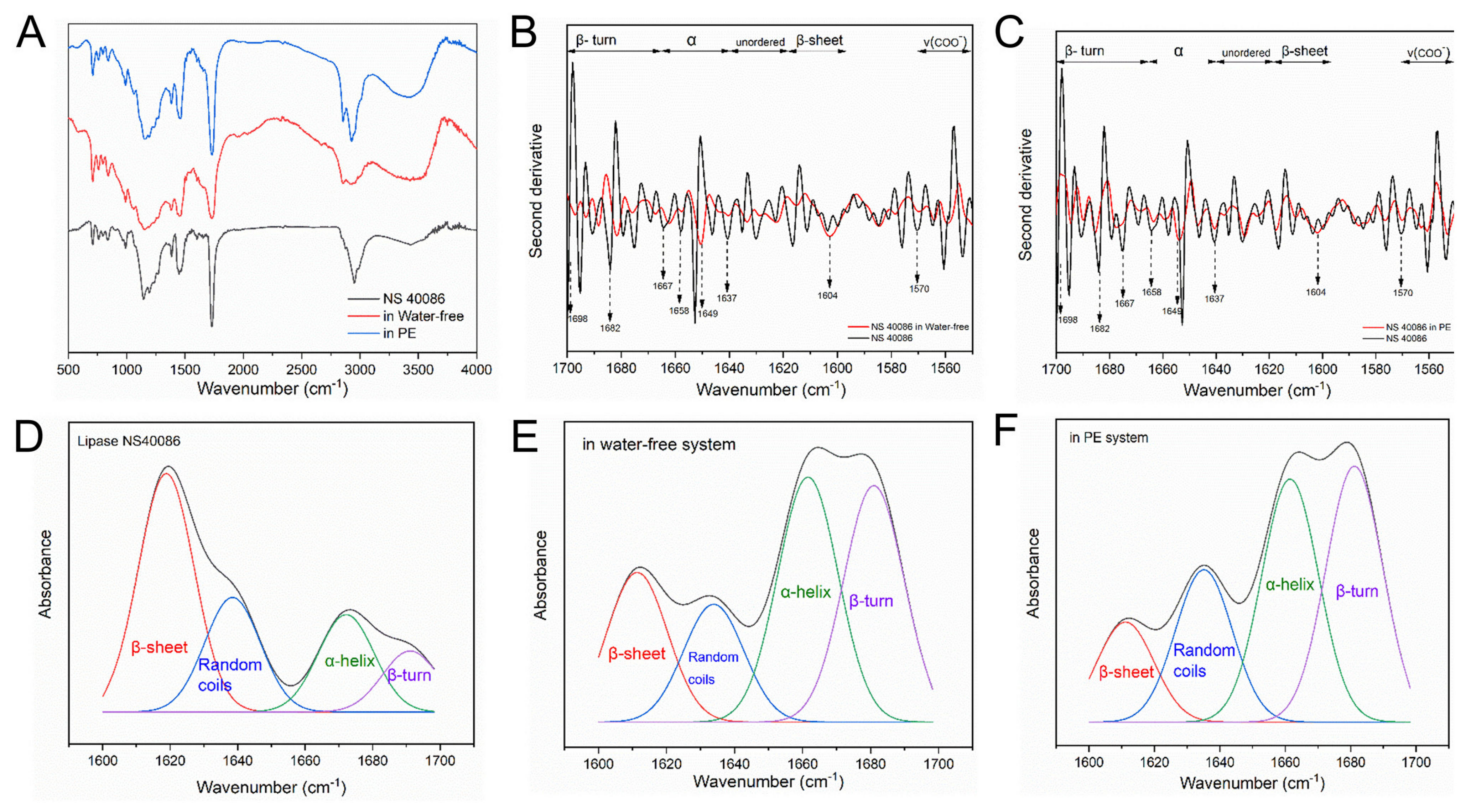
(**A**) FTIR spectra of NS 40086 before the reaction and after the reaction in the water-free system and the PE system. (**B**) Second derivative of the amide I band for NS 40086 before the reaction and after the reaction in the water-free system. (**C**) Second derivative of the amide I band for NS 40086 before the reaction and after the reaction in the PE system. Secondary structural changes of (**D**) lipase NS 40086, (**E**) biocatalyst in water-free system and (**F**) biocatalyst in PE system based on modeled multicomponent peak area by Gaussian multicomponent fitting.

To further confirm the important role of the oil–water interface in improving lipase activity in the PE system, an FT-IR structural analysis of lipase NS 40086 in different reaction systems was performed (Figure 3A–C). Considering that the amide I band at 1700–1550 cm^−1^ is the most sensitive region of the secondary structure information of lipase, the secondary conformational change in the lipase can be reflected by the amide I absorption patterns. The second derivative of the FT-IR spectra of lipase in different systems was calculated with an FT-IR tool (Figure 3D–F). As reported previously [21], the stretches characteristic of the β-sheet structure were located at 1610–1640 cm^−1^, the α-helix structure at 1640–1670 cm^−1^, and the β-turn structure and the random coil structure at 1660–1700 cm^−1^. The bands centered at 1550–1570 cm^−1^ were assigned to the deprotonated υ(COO−) of the side chains of amino acids glutamic and aspartic acid. As shown in Table S3, the relative intensity of the β-sheet showed a sharp decline compared with lipase NS 40086 before use, whereas the relative intensity of the α-helix increased in all cases from 18.25% in the original NS 40086 to 32% in lipase after the reaction. In the case of random coils, the relative intensity in two different systems exhibited an obvious discrepancy. The spectra of the lipase in the Pickering emulsion system showed an intensity of 20.27%, which was similar to that of the original NS 40086 (21.49%) but higher than that of the lipase in the water-free system (15.74%). It indicated that a part of the β-sheet of the lipase transformed into an α-helix structure when the lipase was located at the oil–water interface. This conformational change might be attributed to the transition of the lid from closed to open, which was consistent with previous studies [21,22,23].

### 2.4. Molecular Dynamics Simulation

**Figure 4:**
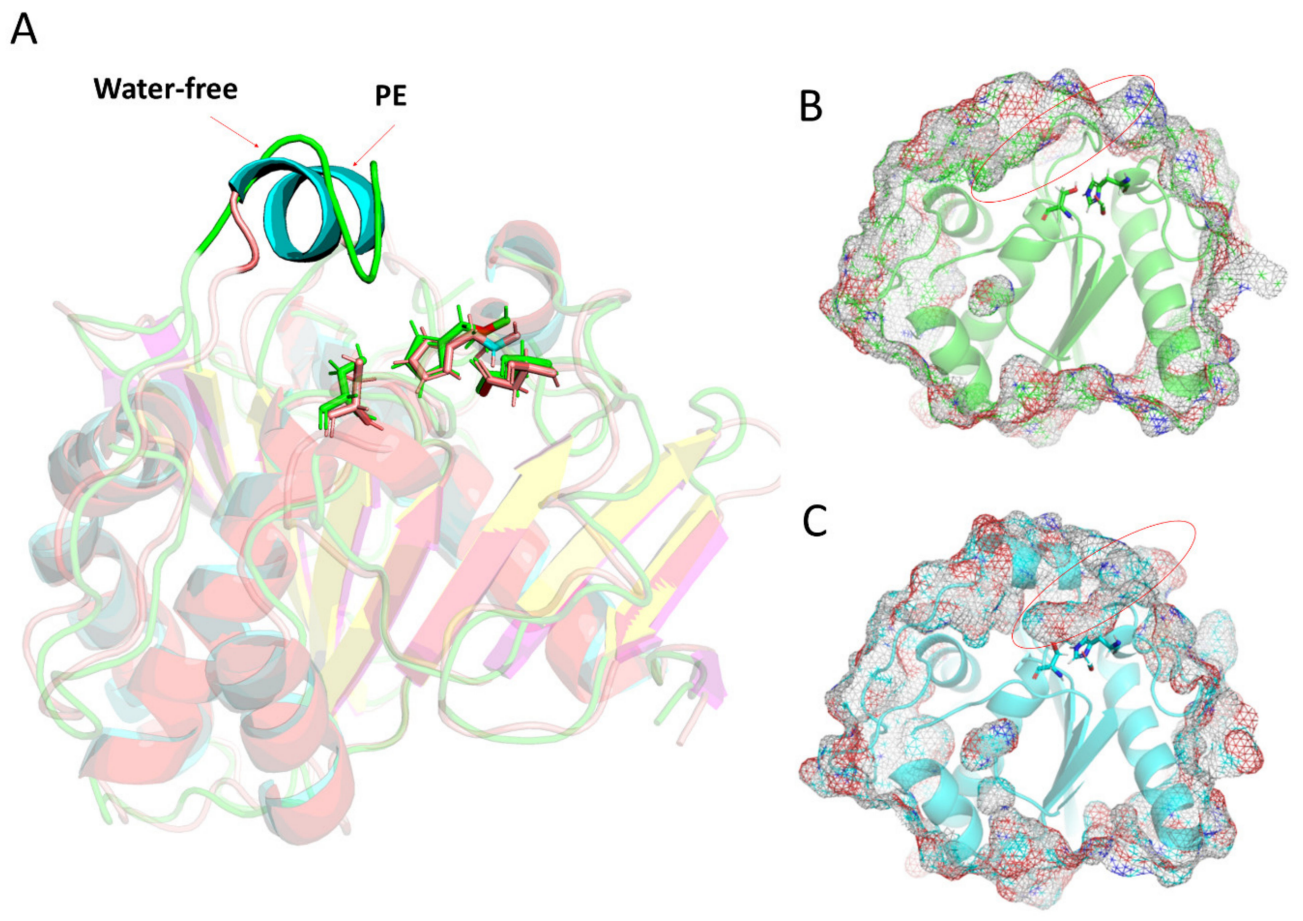
Surface and cartoon representation of NS 40086. (**A**) The residue regions Ser144, Asp203, and His257 are given in sticks (green and orange). The lid residue region Arg80-Val97 is given in green (water-free system) and cyan (PE system). (**B**) State of the active site and lid structure of lipase in the water-free system, and the tunnel (circled in red) is reshaped and bigger, where substrate binding site is located. (**C**) Location and state of the active site and lid structure of lipase in the PE system. The active site pocket has managed to survive and maintain its shape with the lid (circled in red).

**Figure 5:**
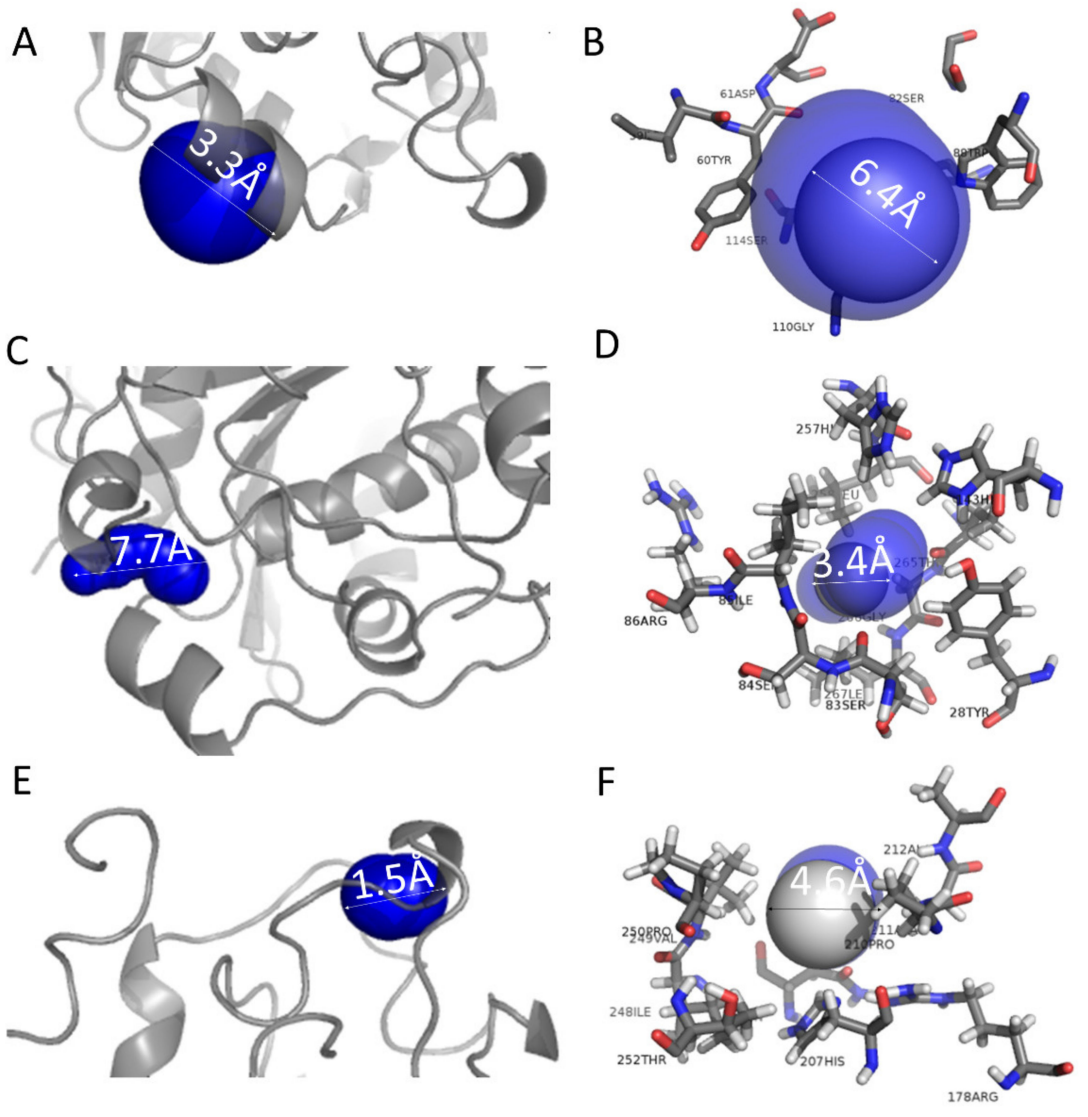
Tunnel 1 and bottleneck residues of lipase (PDB ID:3TGL): (**A**,**B**) initial state, (**C**,**D**) in the water-free system, and (**E**,**F**) in the PE system (Blue: tunnel 1; licorice: bottleneck residues).

As mentioned below, the catalytic performance in the PE reaction system was much higher than that in the water-free system. We inferred that it might have resulted from the lipase conformation change via the structure characterization by FT-IR. To investigate the lipase conformational change at the molecular level, an MD simulation was performed with 1 ns under the optimized conditions. The figures of the RMSD and several snapshots of the MD simulation were used to illustrate the lipase conformation in the two different reaction environments. The RMSD is the measure of the deviation from the crystal structure of a protein, indicating enzyme stability in a system. As shown in Figure S2, the average RMSD value was only 0.1 Å, suggesting that the lipase was stable in the simulation and that the results were acceptable. Figure 4 exhibits the difference in the lipase NS 40086 in the two reaction systems, especially focusing on the active site and the state of the lid. As depicted in Figure 4A, the state of the lid was different in the water-free system (colored in green) and in the PE system (colored in cyan). In the first 1 ns, the lid remained in its flexible α-helix structure in the PE system, while it changed to the random coil structure in the water-free system. Furthermore, the active site pocket was altered or even shrunk by the conformational transformations of the lid in the water-free system (Figure 4B), and the tunnel (circled in red) was destroyed, where the substrate binding site was located. In comparison, the active site pocket and tunnel managed to survive and maintained their shape with the lid in the PE system (Figure 4C), indicating a more seamless and effective binding to the substrate. To figure out the real state of the tunnel in each system, a tunnel analysis was conducted using the Caver web online server. A shorter tunnel brings the center of the pockets close to the surface and shortens the distance between the binding site and the substrate. In the water-free system, the length of the tunnel was increased from 3.2 Å to 7.7 Å, and the bottleneck was decreased from 6.4 Å to 3.4 Å (Figure 5A,B). It suggested that the active site pocket was changed and reformed, which was consistent with the result of the MD simulation. A longer tunnel brings the center of the active pockets far from the surface and makes it harder for the substrate to enter. In the PE system, the lipase showed the shortest tunnel (1.7 Å), with a bottleneck radius of 4.6 Å (Figure 5C). The shortest tunnels have large, open, and easily accessible catalytic sites, which make the active pockets span to the surface. The curvature of the tunnel in our case was closer to 1, indicating a straighter and easier-to-pass tunnel. This lipase was in a closed form in the initial state, with the highest curvature value of 1.6. In the PE system, the curvature value decreased to 1. This indicated that the oil–water interface facilitated the lid and tunnel opening and made the latter into an easier-to-pass tunnel, contributing to substrate binding to the active site.

### 2.5. Reaction Kinetics

**Figure 6:**
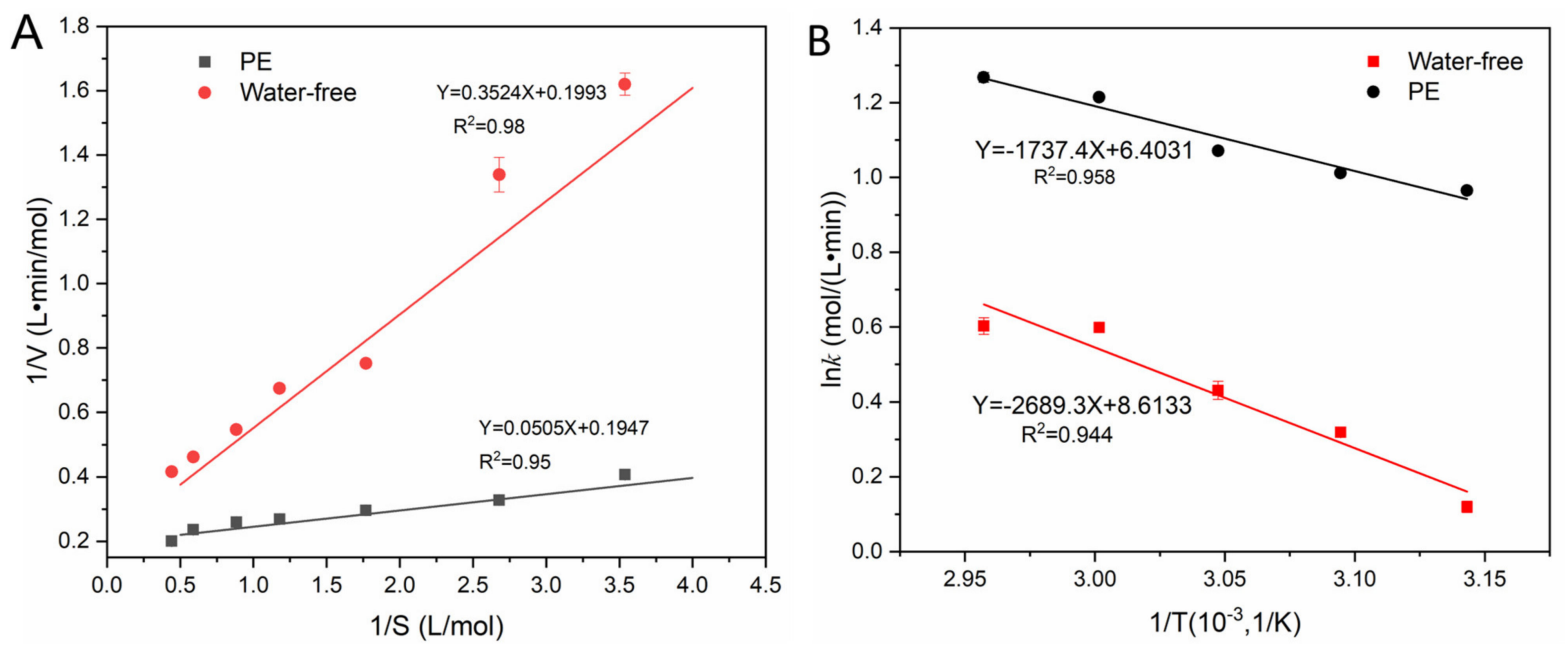
(**A**) Lineweaver–Burk plot of the reciprocal of the initial rate and the MLCT content. (**B**) Arrhenius plot of 1/T versus ln *k* of temperature and the MLCT content.

The kinetic parameters were obtained using the Michaelis–Menten model and the Arrhenius law to provide a more intensive comparison of the catalytic performance in the water-free reaction system and the PE system. According to the Lineweaver–Burk Equation (1), the plot on behalf of 1/*V* versus 1/S showed good linearity at different substrate concentrations (Figure 6A), and the kinetic parameters are summarized in Table 1. The *K_m_* value is regarded as a symbol of the enzymatic affinity for the substrate, denoting that a lower *K_m_* value represents a higher affinity, better substrate binding, and, consequently, a superior catalytic performance. The *K_m_* value of the Pickering emulsion system was 6.8 times lower (0.259 mM) than that of the water-free system. This indicated that the lipase in the PE system was more accessible to the substrate and had a deeper affinity for the substrate. This was coherent with the results obtained using MD simulations and Caver. The shorter a tunnel is, the easier it is for a substrate to bind to the lipase. The tunnel analysis revealed that the lipase tunnel in the PE system was shorter (1.7 Å), with a larger bottleneck radius of 4.6 Å (Figure 5C) and a curvature closer to 1, indicating a straighter and easier-to-pass tunnel. Thus, this lipase conformation boosted the binding of the substrate to the lipase. The *V*_m_/*K*_m_ value is often considered an indicator of enzyme performance. The constant *V*_m_/*K*_m_ value of the Pickering emulsion system was 6.97 times higher than that of the water-free system, indicating that the oil–water interface highly boosted the catalytic efficiency of the lipase. These findings highlight the significance of the oil–water interface and the superior advantage of the PE system in the enzymatic synthesis of MLCT-rich structured lipids.

As it is pointed out in Figure 1D, the effect of the temperature on the MLCT content was investigated. Again, the linear Arrhenius regression plots of ln *k* versus 1/T for the two reaction systems are exhibited in Figure 6B, and the corresponding kinetic parameters are calculated with Arrhenius Equation (3). The activation energy (E_a_) is the indicator of the ease with which a reaction proceeds. A lower E_a_ value stands for a more effective catalytic performance under the same conditions. The E_a_ of the Pickering emulsion system was 14.45 kJ mol^−1^, which was much lower than that of the water-free system (22.36 kJ mol^−1^). This might be explained by the fact that the greater oil–water interface in the PE system facilitated the opening of the lipase lid, allowing for easier access to the catalytic site and, thus, activating the enzyme.

### 2.6. Triacylglycerol Compositions in Two Systems

In the above sections, the content of MLCT in the two systems was determined by HPLC-ELSD. However, each TAG molecular species, like the composition of TAGs, was unknown. To provide better insight into the TAG molecules in the two reaction systems, the products were detected by UPLC-Q-TOF-MS, including the composition and the relative content of the TAGs. In summary, there were about 36 species of TAGs in the water-free reaction system and 38 TAG species in the PE system. Table 2 compares the TAG compositions in the two reaction systems. Most of the TAG products in the water-free system were similar to those in the PE system, but their relative content displayed a significant difference. The three primary TAG species in the water-free system were 8:0/18:1/10:0 (13.13%), 8:0/18:0/8:0 (11.06%), and 18:1/8:0/18:1 (10.83%). In the PE system, the primary TAG products were the same as those in the water-free system, with 8:0/18:1/10:0 and 8:0/18:1/8:0 comprising 13.53% and 10.74% of the total TAG content, respectively. On the basis of the chain length of the fatty acids and their distribution, the obtained TAG products could be divided into MML, MLL, LML, and MLM. In the products, there were about 42.37% and 42.63% TAGs with an MML-type or an MLM-type in the water-free system and the Pickering emulsion system, respectively. The most abundant MLCT of the MLM-type was 8:0/18:1/10:0 in both systems. As for the LML-type or the MLL-type, they accounted for 30.38% and 32.48% of the total TAG content in the water-free system and the PE system, respectively. The major TAG of the LML-type was 18:1/8:0/18:1, which accounted for 10.83% and 9.34%, respectively. Interestingly, there were three TAG molecules in the PE reaction system not present in the water-free reaction system, which were 16:0/8:0/18:2 (1.18%), 10:0/18:1/18:0 (1.06%), and 18:3/10:0/18:1 (0.57%).

When comparing the TAGs in the substrate (Table S2) and its product in the products of the reaction (Table 2), the conversion was calculated based on the change in TAG content in the LCT, because the other substrate, MCT, was in excess. The conversion was 90.26% in the PE system and 89.04% in the water-free system. Unfortunately, there were free fatty acids, diacylglycerols, and monoglycerides in the products, especially in the PE system (Table S4). The final yield of TAGs was only 63% after purification. Thus, the yield of MLCT was 46% in the PE system. Further work should be carried out about inhibiting this side reaction.

**Figure 7:**
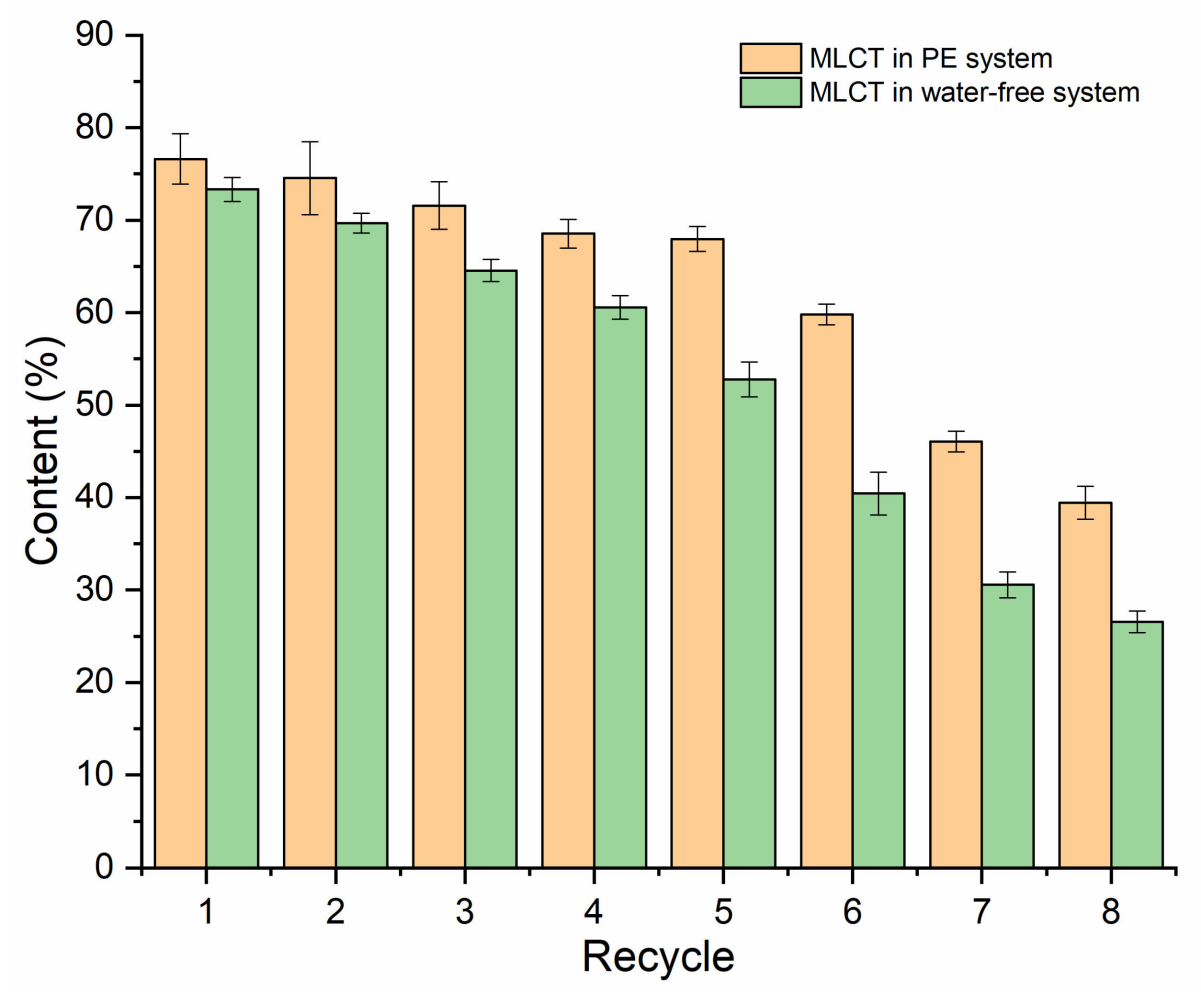
Reusability of NS 40086 in 10 repeated cycles under standard conditions. Reaction conditions: substrates ratio, 1:1; enzyme loading, 10%; reaction time, 2 h; and PBS dosage, 3%.

The reusability of particle catalyst in a Pickering emulsion is one of the most necessary features for the latter’s practical applications. Therefore, the reusability of NS 40086 in these two systems was analyzed for 10 repeated cycles under standard conditions. Lipase NS 40086 achieved an MLCT content of 40% after 10 cycles in the PE system (Figure 7), while it gained an MLCT content of 20% in the water-free system. This decrease in lipase activity might have resulted from enzyme denaturation during the emulsification process. Future work should be carried out about the exploration of emulsification methods or new immobilized lipase preparations with better reusability.

In summary, we developed a highly effective PE system for the enzymatic synthesis of MLCT-rich structured lipids. Lipase NS 40086 served simultaneously as the catalyst and as a solid emulsifier to stabilize the Pickering emulsion. Benefitting from the sufficient oil–water interface, the resulting PE system achieved 77.5% MLCT content within 30 min, which was 26% higher than that of the water-free system under the following optimal conditions: substrates weight ratio, 1:1; enzyme loading, 10% (*w*/*w*); reaction time, 2 h; and PBS dosage, 3% (*w*/*w*). The increase in the *K_m_* value (0.259 mM) in the PE system was 6.8 times lower than that in the water-free system (1.768 mM), and the activation energy (14.45 kJ mol^−1^) in the PE system was 1.6 times lower than that in the water-free system (22.36 kJ mol^−1^). The kinetic parameters implied that the oil–water interface enhanced the binding between the substrate and the lipase, thus boosting catalytic efficiency, as evidenced by the MD simulation and tunnel analysis performed. The structural characterization using FT-IR revealed that the β-sheet of the lipase was transformed into an α-helix structure when the lipase was located at the oil–water interface, a phenomenon which might be attributed to the transition of the lid from closed to open. Hence, such an excellent catalytic performance in MLCT synthesis in the PE system might be ascribed to the facilitation of lid opening by its abundant oil–water interface. This study will pave the way for the advancement of progress in PE as a sustainable and high-efficiency platform for the enzymatic synthesis of functional lipids.

## 3. Materials and Methods

### 3.1. Materials

The MCT and LCT were purchased from Youchuang Industrial Co., Ltd. (Shanghai, China) and local supermarkets (Wuxi, China), respectively. Lipase NS 40086 (immobilized lipase from *Rhizomucor miehei*, 275 IUN/g, particle form) was obtained from Novozymes (Beijing, China). The IUN is equal to the trans-esterification unit and defined as the amount of enzyme activity generating 1 μmol of propyl laurate per minute under defined standard conditions. The mixed fatty acid methyl esters standard and lipase from porcine pancreatic (type Ⅱ) were acquired from Sigma-Aldrich Chemical Co., Ltd. (Shanghai, China). HPLC-grade solvents, including hexane, isopropanol acetonitrile, and methanol were purchased from J&K Scientific Co., Ltd. (Beijing, China). Other analytical reagents were supplied by Sinopharm Chemical Reagent Co., Ltd. (Shanghai, China).

### 3.2. Lipase-Catalyzed Trans-Esterification of MCT and Camellia Oil

#### 3.2.1. Construction of Pickering Emulsion Reaction System

The substrates (MCT and LCT), the lipase NS 40086, and the PBS solution were vigorously shaken for 5 min with a vortex to construct the PE system. The addition of PBS (50 mM pH 7.0) varied in its amount from 0.5% to 5.0% (relative to the mass of the substrates). The effect of the pH value of the PBS solution on the conversion was evaluated in the pH range from 5.0 to 9.0 with a substrate ratio of 1:1 (MCT to camellia oil), a temperature of 60 °C, and an enzyme dosage of 10% for 4 h. At the end of the reaction, the samples were subjected to centrifuging at 8000 rpm for 6 min for phase separation. The upper layer of the oil phase was collected for analysis. The solid catalyst (NS 40086) was sedimented at the bottom of tube, washed with *n*-hexane, and dried for the next reaction cycle.

#### 3.2.2. Optimization of Lipase-Catalyzed Trans-Esterification

Lipase-catalyzed trans-esterification was carried out in a 25 mL double-jacketed beaker with magnetic agitation at 350 rpm. The reaction temperature was maintained by a thermostatic water bath. The reaction mixture contained MCT and camellia oil in a water-free catalytic system. The effect of the mass ratio of the camellia oil to the MCT (0.5:1, 1:1, 1.5:1 and 2:1) and that of the enzyme dosage (4–12%) were investigated under a temperature of 60 °C for 4 h.

### 3.3. Enzymatic Kinetic Study

The enzymatic kinetic parameters of these two reaction systems were evaluated using the Michaelis–Menten model. The apparent kinetic parameters were determined at the different concentrations of camellia oil tested, ranging from 0.5 to 4 mM, for 3 min under the optimum conditions. The Michaelis constant (*K*_m_, mol/L) and maximum reaction rate (*V*_m_, mol L^−1^ min^−1^) were calculated by fitting the data with the Lineweaver–Burk Equation (1):

(1) $$\frac{1}{v_{0}}=\frac{K_{m}}{v_{m}}\frac{1}{\left[S\right]}+\frac{1}{v_{m}}$$

where *V*_m_ is denoted as the maximum reaction rate, *V*_0_ is the initial reaction rate, [S] is denoted as the initial concentration of camellia oil, and *K*_m_ is the Michaelis–Menten constant.

The temperature dependence of the kinetic parameters in these two reaction systems were assayed after incubation at 45, 50, 55, 60, and 65 °C for 3 min under the optimum conditions using the Arrhenius Equations (2) or (3):

(2) $$k=Aexp\left(-\frac{E_{a}}{RT}\right)$$

(3) $$\ln k=lnA-\frac{E_{a}}{RT}$$

where *k* is the reaction rate constant and is defined as the initial enzyme reaction rate, *A* is the Arrhenius constant, *E_a_* refers to the activation energy (J/mol), R is the universal gas constant (8.3145 J mol^−1^ K^−1^), and T is the absolute reaction temperature (K). The activation energy *E_a_* could be estimated from the regression line of ln*k* versus 1/T.

### 3.4. Analysis of Reaction Products

#### 3.4.1. Isolation of Reaction Products

The crude products were separated by thin-layer chromatography (TLC, silica gel GF UV-254 plates, 10 cm × 20 cm) using hexane, diethyl ether, and acetic acid as the mobile phase at a ratio of 80:20:1 (*v*/*v*/*v*). The lipid mixture (22 mg) was dissolved in diethyl ether (120 μL), which was spotted onto the dried TLC plates and developed in the tank for about 50 min or longer. Triolein was applied as the TAG standard and run simultaneously alongside the samples for the recognition of the TAG. The developed plates were dried in the air and left for 30 s in a tank of iodine vapor, which could render the lipids visible as brown bands. The TAG band was scraped off and eluted with diethyl ether three times. The separated TAGs were utilized for further analysis. The fatty acids of the substrates and products were converted into the corresponding methyl esters, as described previously [24]. The analysis of *sn*-2 fatty acid composition was performed according to our previous study [25]

The resulting fatty acid methyl esters were determined using an Agilent 7820A gas chromatograph (Agilent, Santa Clara, CA, USA) equipped with a flame ionization detector and a DB-Fast FAME capillary column (30 m × 0.25 mm × 0.25 μm, Agilent Technologies Corp., Santa Clara, CA, USA). The detection conditions were as follows: the high purity nitrogen (99.999%) was set as the carrier gas, with a flow rate of 1 mL/min; the injection volume was 1 μL at a split ratio of 100:1; the injector and detector temperatures were maintained at 250 and 260 °C, respectively; and the oven was held at 80 °C for 0.5 min, programmed to 165 °C at the rate of 40 °C/min and held for 1 min, and then further increased to 230 °C at a rate of 2 °C/min, with a final maintenance time of 2 min. The fatty acid composition was measured according to the peak area of each fatty acid methyl ester relative to the total peak area.

#### 3.4.2. Analysis of Triacylglycerol Composition

The determination of the TAG composition of a product was performed on the Agilent 1260 HPLC system (Agilent, Santa Clara, CA, USA) equipped with an evaporative light-scattering detector (ELSD) and a Lichrospher C18 column (250 mm × 4.6 mm × 5 μm,). The detection conditions were as follows: the mobile phase was composed of (A) methanol, (B) acetonitrile, and (C) isopropanol with a flow rate of 0.8 mL/min; the gradient elution started with 30% A/70% B at 0–5 min, switched to 30% C/70% B at 5–10 min, and increased to 40% C/60% B at 10–45 min, 45% C/55% B at 45–90 min, further raised to 50% C/50% B at 90–100 min, finally decreased to 40% C/60% B at 100–112 min, and ended up with 30% A/70% B at 112–120 min; and the temperature of the ELSD was set to 55 °C, and the gas flow rate was 1.8 mL/min. The extracted TAG samples were dissolved with n-hexane and diluted to 5 mg/mL with an injection volume of 10 μL.

The TAG molecular species were identified by UPLC-Q-TOF-MS (Waters, Milford, MA, USA) as described previously [26,27].

### 3.5. Structural Characterization of Lipase

The microstructure of the Pickering emulsion was observed by means of confocal laser scanning microscopy (CLSM). The water phase was dyed with 20 μL fluorescein isothiocyanate isomer I (FITC-I, 0.02%, *w*/*v*). The staining of lipase NS 40086 followed a previous publication [28]. The solid particles were dispersed into FITC-I (0.02%, *w*/*v*). The mixture was stirred for 12 h at room temperature in the darkness. The labelled particles were collected by centrifugation. Confocal observation was performed on Zeiss LSM 880 with Airyscan (Zeiss, Jena, Germany) with an argon laser operating at a 514 nm excitation wavelength (Nile red) and a 488 nm excitation wavelength (FITC-I).

The chemical structures of the catalysts, including lipase NS 40086 before and after the reaction, were measured using an FT-IR spectrometer (IS10, Nicolet, Madison, WI, USA) equipped with an ATR accessory. The samples were made in a KBr tablet. The FT-IR spectra were recorded from 4000 to 400 cm^−1^ with 32 scans at a resolution rate of 4 cm^−1^. FT-IR spectra data analyses were performed using OMNIC 8.0, following a previous work [23].

### 3.6. Molecular Dynamics Simulation

To study the protein structure at the molecule level, an MD simulation was performed on the GROMAS 2018 software package (http://www.gromacs.org/, accessed on 1 January 2020). The CHARMM36 force field was used for the MD simulation. The crystal structure of lipase NS 40086 (PDB ID: 3TGL) was obtained from the Protein Data Bank as the initial structure in a closed conformation for the simulation. The protein was dissolved in an explicit solvent. Considering that the primary TAGs in the reaction are triolein and tricaprylin, as for the water-free system, 44 triolein molecules (PubChem CID: 5497163) and 47 tricaprylin molecules (PubChem CID: 10850) were added to the simulation box. In the simulation of the PE system, a certain proportion of water molecules (about 9056) was added in the box, the TIP3P water model was selected, and 55 triolein molecules and 39 tricaprylin molecules acted as the oil phase. The model was placed in a cubic box. Energy minimization was performed using the steepest descent method. Furthermore, the system was first equilibrated for 100 ps under an isothermal-isochoric (NVT) system and then for 100 ps under an isothermal-isobaric (NPT) system using a leap-frog integrator and reached the corresponding simulated temperature (298.15 K) and pressure (0.1 MPa). The balanced system was simulated under the NPT system for 5 ns of all-atom molecular dynamics. The LINCS algorithm was used for all the key constraints in the system. Long-range electrostatic interactions were calculated using the Particle Mesh Ewald method with a time step of 2 fs. The root mean square deviation (RMSD) was studied to make sure that the protein structure was fully relaxed. Protein structure characterization and pertinent images were obtained using standard tools supplied by the Gromacs package, PyMol (Schrödinger, Inc., LLC, New York, NY, USA), VMD (University of Illinois, Champaign, IL, USA). The Caver web online server was used to conduct the tunnel analysis of lipase in the water-free system and the PE system [29].

### 3.7. Statistical Analysis

The data represent the mean ± standard deviation. All the experiment data were collected in triplicate. The analysis of the data was performed with a one-way ANOVA, and their significant differences (*p* < 0.05) were tested with Duncan’s multiple-range tests on IBM SPSS Statistics 20 (SPSS, Chicago, IL, USA).

## Figures and Tables

**Table 1:** Comparison of the kinetic parameters in the water-free system and in the Pickering emulsion system.

	*K*_m_ (mM)	*V*_max_ (mM/min)	*V*_max_/*K*_m_ (min^−1^)
Water-free system	1.768	5.018	2.837
PE system	0.259	5.136	19.802

**Table 2:** Relative content (%) of TAGs in two different reaction systems.

Peak Number	[M + NH_4_]^+^	Composition	Relative Content (%)	
			Water-Free	Pickering Emulsion
1	488.39	8:0/8:0/8:0	2.26 ± 0.06	2.18 ± 0.03
2	516.4	8:0/10:0/8:0	4.19 ± 0.82	4.06 ± 0.83
3	544.45	8:0/10:0/8:0	4.80 ± 1.13	3.99 ± 0.67
4	572.48	10:0/10:0/10:0	1.70 ± 0.22	1.57 ± 0.02
5	624.5	8:0/18:2/8:0	2.23 ± 0.06	2.11 ± 0.03
6	600.52	8:0/16:0/8:0	2.63 ± 0.31	2.31 ± 0.32
7	626.5	8:0/18:1/8:0	11.26 ± 1.46	10.74 ± 2.01
8	652.5	10:0/18:2/8:0	1.15 ± 0.06	2.29 ± 0.18
9	628.5	10:0/16:0/8:0	0.68 ± 0.01	1.01 ± 0.03
10	628.5	8:0/10:0/16:0	0.37 ± 0.00	nd
11	654.5	8:0/18:1/10:0	13.13 ± 1.49	13.53 ± 2.88
12	622.5	8:0/18:3/8:0	0.22 ± 0.01	0.21 ± 0.01
13	680.5	10:0/18:2/10:0	1.51 ± 0.03	1.46 ± 0.06
14	656.5	10:0/16:0/10:0	1.24 ± 0.02	1.22 ± 0.26
15	650.5	10:0/18:3/8:0	0.25 ± 0.00	0.24 ± 0.00
16	682.5	10:0/18:1/10:0	6.96 ± 1.83	6.33 ± 1.41
17	684.5	10:0/18:0/10:0	0.74 ± 0.45	1.18 ± 0.03
18	736.6	16:0/18:2/8:0	1.28 ± 0.02	1.16 ± 0.01
19	758.6	8:0/18:2/18:3	0.29 ± 0.01	0.27 ± 0.00
20	788.6	18:2/10:0/18:2	0.61 ± 0.00	2.64 ± 0.03
21	762.6	18:1/10:0/18:2	3.68 ± 0.06	3.16 ± 0.36
22	738.6	16:0/8:0/18:1	3.77 ± 0.83	3.46 ± 0.06
23	764.6	18:1/8:0/18:1	10.83 ± 1.92	9.34 ± 1.95
24	760.8	18:2/8:0/18:2	1.21 ± 0.03	1.31 ± 0.02
25	790.6	18:2/10:0/18:1	1.54 ± 0.02	1.56 ± 0.03
26	792.7	18:1/10:0/18:1	5.09 ± 0.96	4.50 ± 0.87
27	766.6	18:1/10:0/16:0	1.32 ± 0.02	1.22 ± 0.44
28	896.7	18:2/18:2/18:2	1.09 ± 0.36	1.16 ± 0.21
29	872.7	18:2/16:0/18:2	0.74 ± 0.01	0.69 ± 0.01
30	794.7	18:1/18:0/10:0	0.76 ± 0.00	1.05 ± 0.03
31	898.7	18:2/18:1/18:2	1.52 ± 0.01	1.51 ± 0.06
32	876.8	18:1/16:0/18:1	1.78 ± 0.03	1.46 ± 0.02
33	902.8	18:1/18:1/18:1	5.46 ± 0.32	4.25 ± 0.06
34	900.8	18:1/18:2/18:1	2.64 ± 0.62	2.16 ± 0.34
35	874.8	18:2/18:1/16:0	1.01 ± 0.04	1.08 ± 0.03
36	904.8	18:1/18:0/18:1	0.07 ± 0.00	0.79 ± 0.01
38	736.6	16:0/8:0/18:2	nd	1.18 ± 0.03
39	794.7	10:0/18:1/18:0	nd	1.06 ± 0.00
40	788.6	18:3/10:0/18:1	nd	0.57 ± 0.01

nd means not detected.

## Data Availability

Data are contained within the article.

## Supplementary Materials

The following supporting information can be downloaded at: https://www.mdpi.com/article/10.3390/molecules29040915/s1, Table S1 Fatty acid compositions (area%) of MCT and camellia oil; Table S2 Triacylglycerol composition of MCT and camellia oil; Table S3 Fraction of secondary structures for Lipase NS40086 before use and after use in water-free system and Pickering emulsion system; Table S4 Relative content (%) of DAGs, MAGs, and FFAs in two different reaction systems; Figure S1 (A) Effect of substrate weight ratio on the MLCT content. Reaction conditions: enzyme loading, 10%; temperature, 60 °C; reaction time, 2 h. (B) Effect of pH value on the MLCT content. Reaction conditions: enzyme loading, 10%; temperature, 60 OC; reaction time, 2 h; substrate ratio, 1:1 (*w*/*w*); Figure S2 RMSD of activity site structure of lipase in (A) water-free system (B) PE system.
