# Supplementary material for: Enzymatic Synthesis of Structured Lipids Enriched with Medium- and Long-Chain Triacylglycerols via Pickering Emulsion-Assisted Interfacial Catalysis: A Preliminary Exploration
Source: Molecules. 2024 Feb 19;29(4):915. doi: 10.3390/molecules29040915 (PMC10893273; doi:10.3390/molecules29040915)
Supplement: Supplementary file 1 [file molecules-29-00915-s001.zip › molecules-2840491-supplementary.pdf]

Table S1 Fatty acid compositions (area%) of MCT and camellia oil

Fatty acids	MCT		Camellia oil	
	Total	Sn-2	Total	Sn-2
8:0	55.11±0.02	54.02±0.16	nd	nd
10:0	44.89±0.01	45.98±0.18	nd	nd
16:0	nd	nd	8.77±0.01	4.12
18:0	nd	nd	2.26±0.02	1.81
18:1	nd	nd	80.16±0.18	72.72
18:2	nd	nd	6.34	2.68
18:3	nd	nd	0.18	0.09
20:0	nd	nd	0.33	0.04
20:1	nd	nd	1.15	14.9

Values presented as means of triplicates ± standard deviation.

Table S2 Triacylglycerol composition of MCT and camellia oil

ECN[a]	Composition	MCT	Camellia oil
24	8:0/8:0/8:0	24.95±0.50	nd
26	8:0/10:0/8:0	40.00±0.70	nd
28	8:0/10:0/8:0	29.05±0.35	nd
30	10:0/10:0/10:0	5.95±0.21	nd
44	18:1/18:3/18:1	nd	0.49±0.03
44	18:1/18:2/18:1	nd	1.29±0.18
46	18:2/18:1/16:0	nd	4.38±0.06
46	18:1/18:2/18:1	nd	10.84±0.22
48	18:1/18:1/18:1	nd	58.70±0.00
48	18:1/16:0/18:1	nd	19.58±0.18
50	18:1/18:0/18:1	nd	4.08±0.03
50	18:1/20:1/18:1	nd	0.87±0.04

[a] ECN, equivalent carbon number. (ECN = acyl carbon number − 2 × double bond number).

Table S3 Fraction of secondary structures for Lipase NS40086 before use and after use in water-free system and Pickering emulsion system.

Lipase in	β-sheet (%) (1600-1640 cm^{-1})	Random coils (%) (1640-1650 cm^{-1})	α-helix (%) (1650-1660 cm^{-1})	β-turn (%) (1660-1700 cm^{-1})
NS 40086	44.76	21.49	18.25	15.50
In water-free	19.98	15.74	32.70	31.57
In PE	13.32	20.27	32.35	34.05

Table S4 Relative content (%) of DAGs, MAGs, and FFAs in two different reaction systems

Peak number		Type	Composition	Relative content (%)	
				water-free	Pickering emulsion
No.	[M+Na]+	DAG			
1	367.4	Cy-Cy	8:0/8:0	3.56	0.16
2	395.4	Cy-Ca	8:0/10:0	7.84	0.25
3	423.4	Ca-Ca	10:0/10:0	2.54	1.04
4	503.15	Cy-L	8:0/18:2	4.15	1.92
5	479.5	Cy-P	8:0/16:0	0.67	0.28
6	505.1	Cy-O	8:0/18:1	17.32	7.07
7	531.5	Ca-L	10:0/18:2	3.64	2.41
8	507.5	P-Ca	16:0/10:0	2.05	1.38
9	533.5	Ca-O	10:0/18:1	12.82	0.48
10	535.5	Ca-S-	10:0/18:0	4.59	0.26
11	615.5	P-L	16:0/18:2	4.39	10.40
12	641.5	L-O	18:2/18:1	7.26	24.50
13	639.5	L-L	18:2/18:2	5.65	19.85
14	645.5	O-S	18:1/18:0	1.7	1.30
15	617.5	O-P	18:1/16:0	4.61	8.82
16	643.8	O-O	18:1/18:1	12.68	12.13
17	529.13	Ln-Ca	18:3/10:0	2	0.45
18	637.5	L-Ln	18:2/18:3	2.52	7.30
No.	[M+Na]+	MAG			
1	241.7	Cy	MAG-8:0	10.15	3.82
2	269.76	Ca	MAG-10:0	11.33	4.67
3	379.97	O	MAG-18:1	15.96	42.90
4	353.92	P	MAG-16:0	14.86	12.60
5	377.95	L	MAG-18:2	15.87	22.79
6	381.98	S	MAG-18:0	16.04	5.77
7	375.95	Ln	MAG-18:3	15.79	7.45
No.	[M–H]–	FFA			
1	143.7	Cy	C8:0	7.29	1.90
2	171.76	Ca	C10:0	12.72	1.06
3	281.97	O	C18:1	36.36	35.77
4	255.92	P	C16:0	31.19	36.30
5	279.95	L	C18:2	4.58	16.74
6	283.98	S	C18:0	1.62	7.86
7	277.95	Ln	C18:3	6.24	0.37

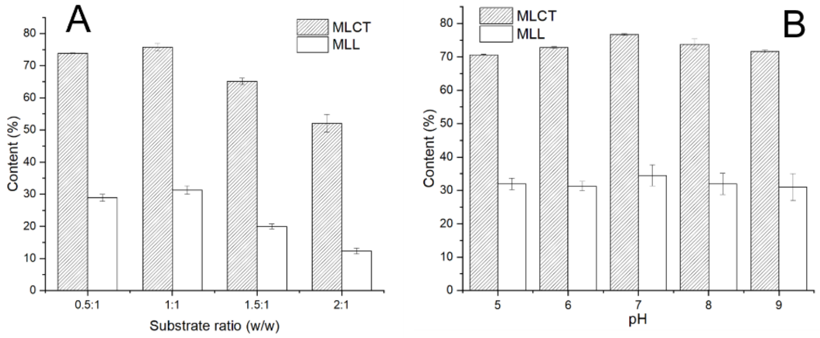

Fig.S1 (A) Effect of substrate weight ratio on the MLCT content. Reaction conditions: enzyme loading, 10%; temperature, 60°C; reaction time, 2 h. (B) Effect of pH value on the MLCT content. Reaction conditions: enzyme loading, 10%; temperature, 60 ^{O}C; reaction time, 2 h; substrate ratio, 1:1 (w/w)

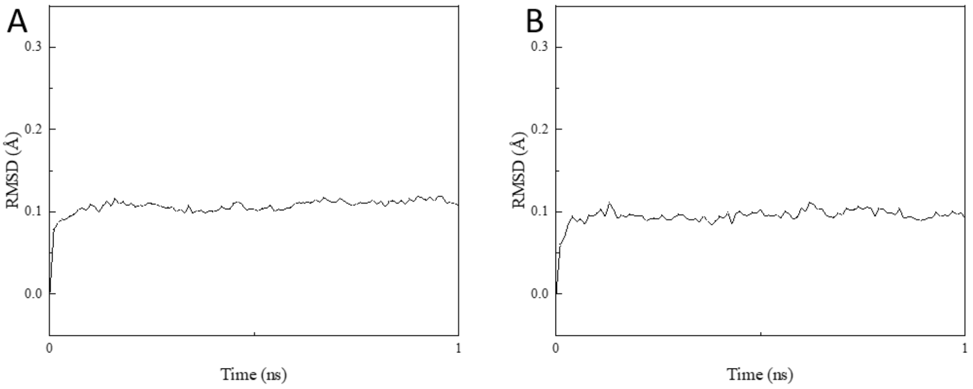

Fig.S2 RMSD of activity site structure of lipase in (A)water-free system (B) PE system.
